# Enhancing the image quality of blue light cystoscopy through green-hue correction and fogginess removal

**DOI:** 10.1038/s41598-023-48882-z

**Published:** 2023-12-06

**Authors:** Shuang Chang, Micha E. Bermoy, Sam S. Chang, Kristen R. Scarpato, Amy N. Luckenbaugh, Soheil Kolouri, Audrey K. Bowden

**Affiliations:** 1 Department of Biomedical Engineering, Vanderbilt University, Nashville, TN 37232 USA; 2 Department of Urology, Vanderbilt University Medical Center, Nashville, TN 37232 USA; 3 Department of Electrical and Computer Engineering, Vanderbilt University, Nashville, TN 37232 USA

**Keywords:** Bladder cancer, Bladder, Imaging and sensing

## Abstract

Blue light cystoscopy (BLC) is a guideline-recommended endoscopic tool to detect bladder cancer with high sensitivity. Having clear, high-quality images during cystoscopy is crucial to the sensitive, efficient detection of bladder tumors; yet, important diagnostic information is often missed or poorly visualized in images containing illumination artifacts or impacted by impurities in the bladder. In this study, we introduce computational methods to remove two common artifacts in images from BLC videos: green hue and fogginess. We also evaluate the effect of artifact removal on the perceptual quality of the BLC images through a survey study and computation of Blind/Referenceless Image Spatial Quality Evaluator scores on the original and enhanced images. We show that corrections and enhancements made to cystoscopy images resulted in a better viewing experience for clinicians during BLC imaging and reliably restored lost tissue features that were important for diagnostics. Incorporating these enhancements during clinical and OR procedures may lead to more comprehensive tumor detection, fewer missed tumors during TURBT procedures, more complete tumor resection and shorter procedure time. When used in off-line review of cystoscopy videos, it may also better guide surgical planning and allow more accurate assessment and diagnosis.

## Introduction

**Figure 1:**
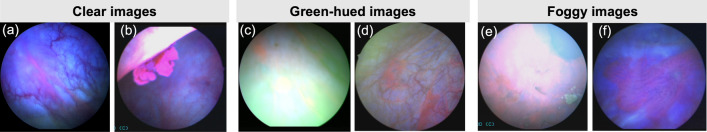
Example clear BLC images (**a**, **b**), green-hue artifacts (**c**, **d**) and foggy artifacts (**e**, **f**).

Cystoscopy, a form of endoscopy specific to the field of urology, is the standard of care to detect and monitor bladder cancer in both clinic and operating room (OR) settings^1^. Due to its ability to provide tumor-specific fluorescent contrast leading to better sensitivity (Fig. 1a, b), blue light cystoscopy (BLC) facilitates better detection and more complete tumor resection than white light cystoscopy (WLC)^2–5^. While high-quality imaging is critical for effective detection and treatment of bladder cancer, many BLC video frames suffer from artifacts that reduce image quality and impede the clinical workflow. For example, the presence of urine can cause the ordinarily blue background to take on a green hue, hindering visualization of tumor contrast in tissue during both in vivo examination as well as offline review (Fig. 1c, d). In cases where the green hue completely obstructs the view, the clinician must remove the cystoscope and allow the urine to drain before they can resume imaging, leading to prolonged OR time. Similarly troubling, many BLC frames appear foggy, which is thought to result from hardware degradation and impurities in the fluid that distends the bladder (Fig. 1e, f). Such foggy frames show sub-optimal clarity, loss of vessel features and diminished fluorescent contrast. Together, green hue and fogginess lead to missed tumors during resection and longer resection time due to challenges in visualization.

Multiple clinical studies have shown that enhancing video quality improves the sensitivity of tumor detection^1,6–8^. While many efforts have been made to improve the imaging quality of WLC videos^1,7^, to our knowledge, no existing strategies have been presented to address the discoloration and fogginess present in BLC imaging. In this work, we propose new image enhancement methods to reduce the green-hue and fogginess artifacts of BLC frames in cystoscopy videos. These methods effectively restore the tumor fluorescence and tissue features lost due to these artifacts and improve overall image clarity while maintaining a realistic appearance for blue light images, leading to better clinical visualization and assessment. Through a survey conducted on six urologists, we show that whereas original blue light frames have sub-optimal clarity and contrast, the enhanced images score higher on visual satisfaction and increase confidence in making clinical decisions. 

## Methods

### Patient recruitment

Under IRB approval from Vanderbilt University (#211206), we recruited 31 patients who were scheduled for a transurethral resection of bladder tumor (TURBT) procedure with Cysview® at Vanderbilt University Medical Center. Informed consents were obtained from all subjects and all methods were performed in accordance with the relevant guidelines and regulations. From each patient, we collected a few short video clips (around 1 min each). In total, we collected 45 videos using a commercial KARL STORZ Blue Light Cystoscopy with Cysview® System. 

### Video pre-processing

During the standard TURBT procedure, clinicians switch intermittently between the WLC and BLC for bladder visualization. Therefore, the collected cystoscopy videos contain both WLC and BLC frames at arbitrary intervals. As the focus of this work is the enhancement of BLC frames, only the blue-light-illuminated portions of the videos were needed. In order to extract the blue light frames from the full cystoscopy video, we developed an automated algorithm to classify each frame as a WLC or BLC frame. In brief, we compared the mean intensities of the blue and green channel data of each frame to the mean grayscale intensity of that frame: frames with high levels of blue and green intensity were classified as BLC frames and saved as images for use in this study. In this work, the terms images and frames are used almost interchangeably.

**Figure 2:**
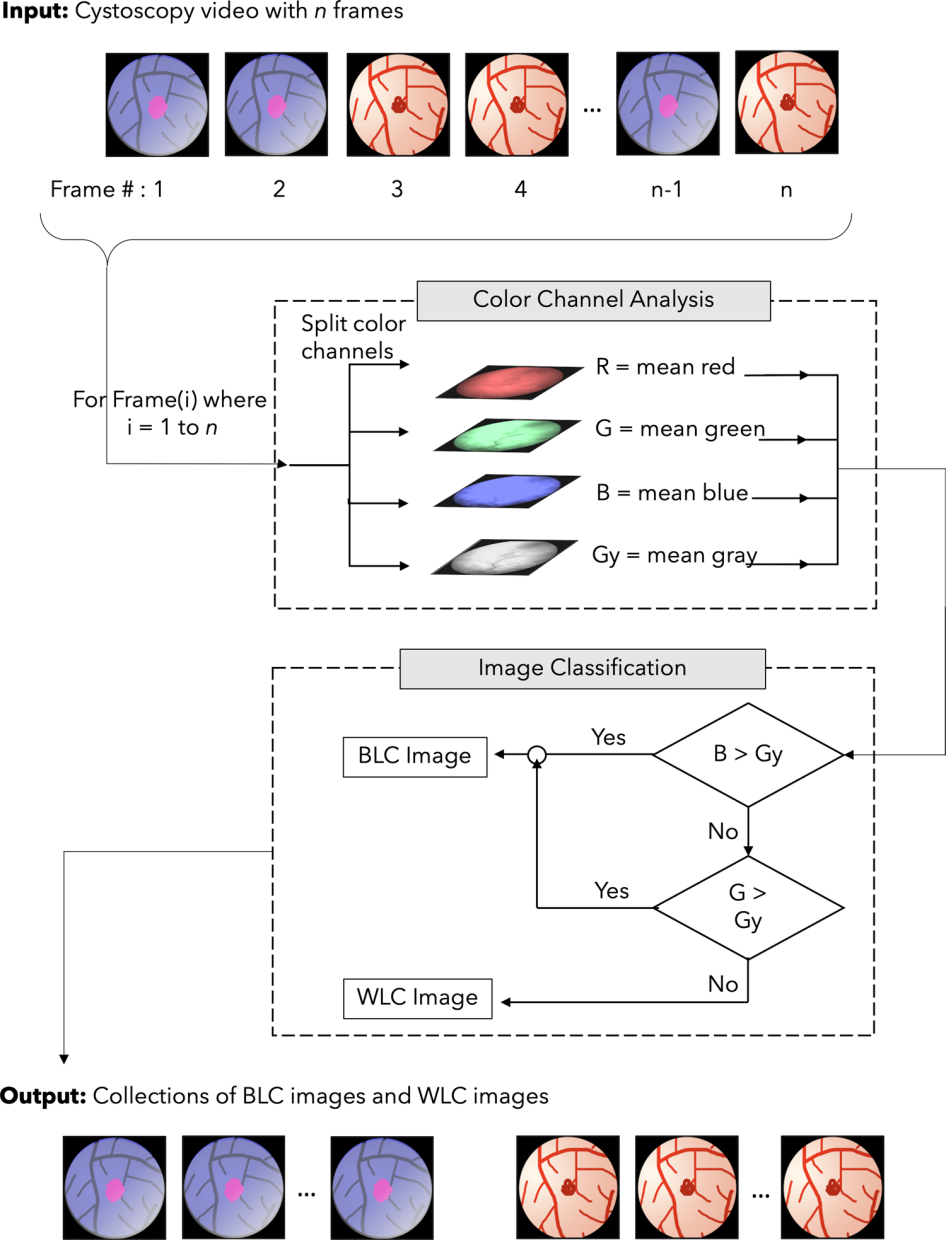
Overview of video pre-processing. “Color Channel Analysis” and “Frame Classification” are sub-steps that take place n times for an input video, where n is the number of frames in a given cystoscopy video. The outputs of this flowchart are collections of WLC images and BLC images.

For frames extracted from cystoscopy videos comprising a mix of WLC and BLC frames, we observed that under blue light illumination, the blue channel (or green channel, in the case of green hue artifact) appears as the background color and shows relatively high intensity; whereas, in WLC, the red channel serves as the background color and dominates in intensity. Our classification algorithm thus comprised two steps. During the “Color-Channel Analysis” step (Fig. 2), we computed the mean RGB color channel and grayscale intensities for each frame and compared the mean blue and green channel intensities against the mean grayscale value. In the “Frame Classification” step, if either the mean blue or mean green intensity was greater than the gray value, the frame was classified as BLC; otherwise, the frame was classified as WLC. The red channel was not used for classification to avoid the situation where a BLC frame containing high fluorescence regions might be falsely recognized as a WLC frame. The end result of this procedure was two sets of video frames: one containing only WLC images and the other containing only the BLC images. The WLC images were then discarded for the remainder of this study.

### Green-hue correction

**Figure 3:**
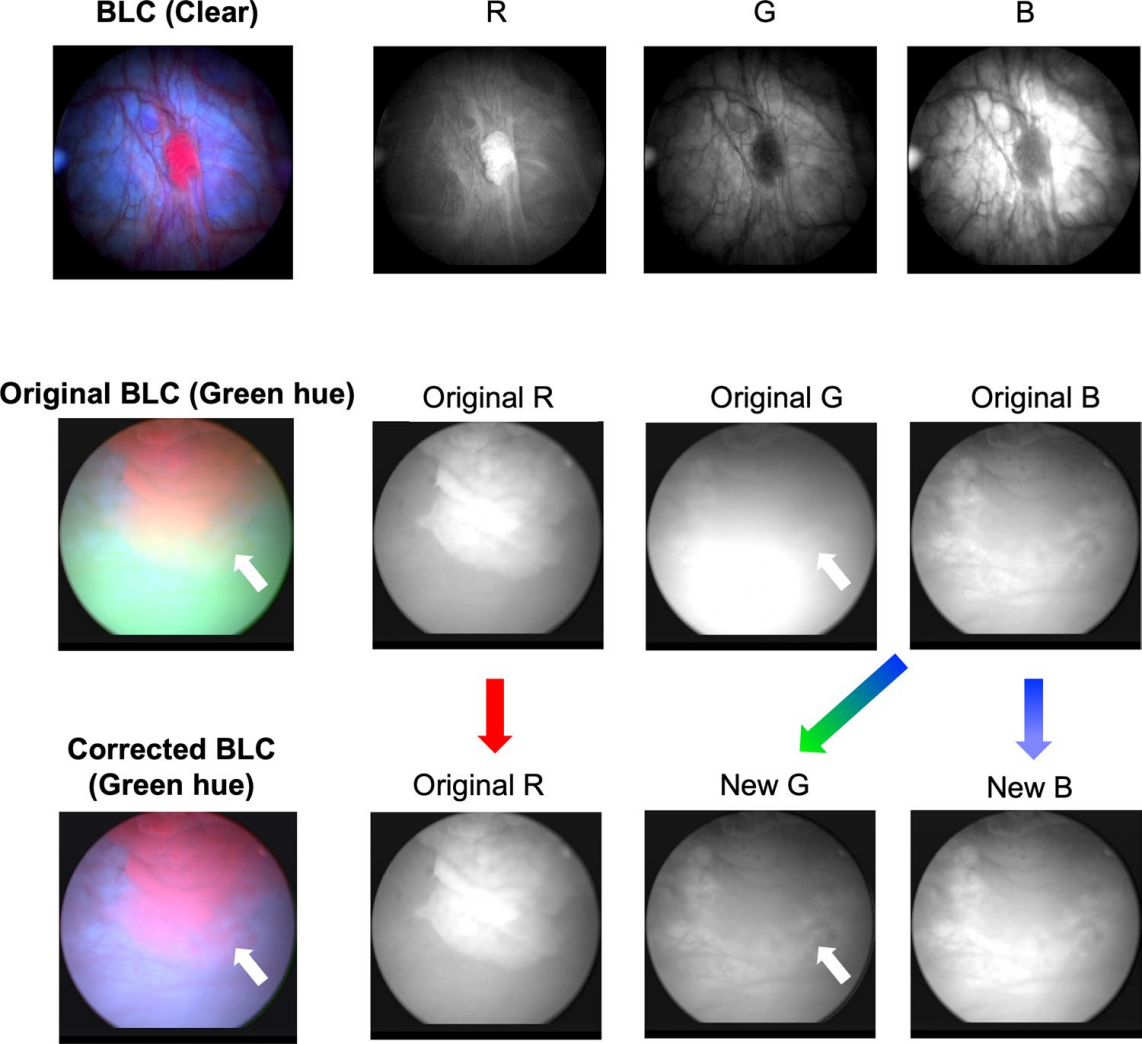
Clear BLC image (top row) and its RGB color channels. Green-hued BLC image before (middle row) and after (bottom row) green-hue correction and their corresponding the RGB color channels. White arrows point at a lost feature in the green-hued image, which is later recovered in the corrected image. Colored arrows indicate color channel operations in the green-hue correction step.

In clear BLC images, the blue channel has the overall highest average image intensity (outside of local fluorescent regions, where the red channel dominates), while the green channel has the lowest average intensity and provides the most vascular contrast (Fig. 3, top row). In the case of green-hued images, the green channel is highly saturated, leading to the loss of tissue features in the green channel. In such cases, we observed that the blue channel serves to better preserve fine tissue features, as shown in Fig. 3 (middle row, original B). Thus, our strategy to address the green-hue artifact was to generate a new green channel whose intensity levels match those of a normal BLC image and whose information content (i.e., vascular contrast) derived from its blue channel.

**Figure 4:**
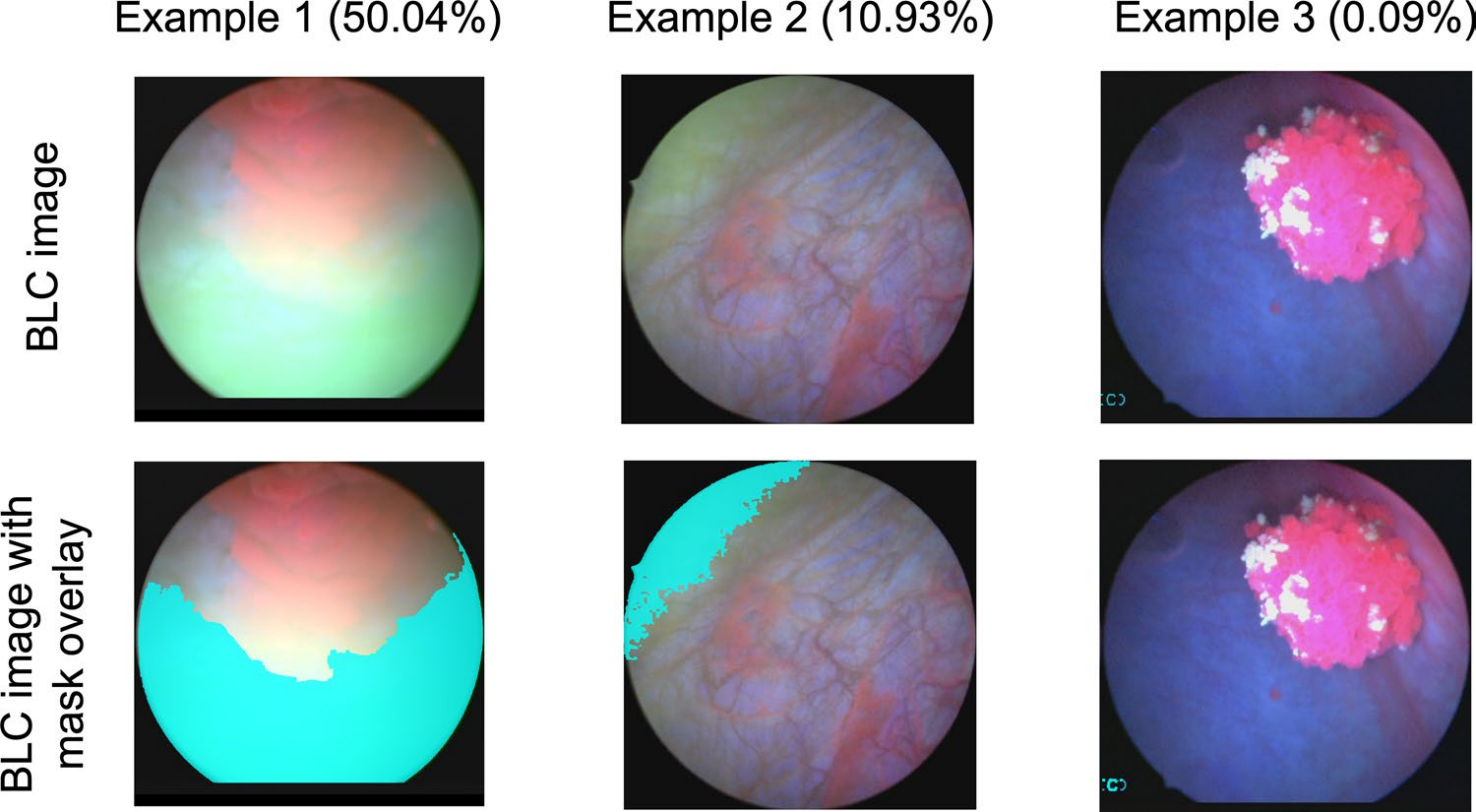
Three example BLC images with green-hue mask overlays shown in cyan. Examples 1 and 2 are classified as green-hue images (green-hue pixel > 5% in the FOV), and example 3 is not classified as a green-hue images.

To detect green-hued images, we used a simple heuristic that considers color-channel intensity. We first identified “green-hue pixels” as those where the green channel intensity was both greater than the blue channel intensity and greater than 105% of the image’s mean grayscale intensity. The resulting binary mask of green-hue pixels was then dilated (kernel size of 10 pixels) and eroded (kernel size of 5 pixels). The largest area of connected components comprised the final green-hue mask, as shown in Fig. 4. The “clear region” of the image is shown as the unmasked pixels in the field of view (FOV). The number of green-hue pixels in the circular FOV was then compared with the total number of pixels in the FOV to determine the green-hue-pixel percentage. An image was recognized as a green-hue image if the percentage of green-hue pixels exceeded 5%.

To correct for green-hue artifact, the original green channel image was discarded (Fig. 3 bottom row), and a new green channel was generated by multiplying the original blue channel intensity by a correction factor whose value was given by the average ratio of the blue-to-green intensity values in “clear” regions of the image (i.e., pixels with low green-channel intensities), not to exceed 0.8. To create a new blue channel for the image, the intensity range of the original blue channel was scaled to match to the range of the original green channel. Next, a median filter with a kernel size of 20 pixels was applied to the new blue channel. The filter served to slightly blur the new blue channel, thereby creating a more realistic BLC image for when the RGB color channels were recombined. No changes were made to the red channel, which carries fluorescence information, when constructing the new image. Because tumor fluorescence is mostly captured by the red channel (tumor tissue lights up as red/pink patches), intensity adjustments made to the green and blue channels did not interfere with tumor fluorescence. 

### Fogginess correction

Despite the frequency of its appearance clinically, the exact cause of fogginess in BLC images has yet to be determined. To address this problem, we took inspiration from work to improve effects of environmental fog in remote sensing^9–11^. Specifically, we leveraged the single-frame visibility restoration strategy introduced by Tarel et al. that introduces a percentage-of-restoration (POR) metric for image enhancement, commonly chosen to be between 90 and 95% for natural scenic or road images^12,13^.

For a given image, fog has two effects on the observed luminance *I* of an object at pixel position [*x, y*] located a distance *z*(*x,y*) away, where the true object luminance is $I_{0} \left( {x, y} \right)$. These effects are embodied in the two terms of the following equation:

1 $$ I\left( {x, y} \right) = I_{0} \left( {x, y} \right)e^{{ - \mu z\left( {x, y} \right)}} + V\left( {x,y} \right). $$

The first effect is attenuation of $$I_{0} $$ by the intervening medium. Here, µ is the extinction coefficient of the medium (i.e., foggy or hazy air). In the case of cystoscopy imaging, *I* is the observed cystoscopy image and $$I_{0} $$ is the corresponding uncorrupted image of the bladder wall, µ is the extinction coefficient of the solution that distends the bladder, and z(x,y) denotes the distance of the bladder wall to the cystoscope (the detector). The second effect is addition of a fogginess factor to the observed image, termed a foggy veil. The veil is thought to describe an increasing luminescence due to atmospheric contribution whose effect is to reduce contrast. It can thus be modeled using the following equation: *V* ($$x,y) = I_{s} \left( {1 - e^{{ - \mu z\left( {x,y} \right)}} } \right)$$, where $$I_{s}$$ is the luminance of the atmospheric light source (i.e., the sky or light source of the cystoscope). The fog itself is thought to be due to the presence of impurities (urine or debris) in the saline.

In the original method by Tarel and Hautière^12^, single-image defogging is achieved by estimating the intensity of the foggy veil *V* from the detected image. By assuming constant brightness of the light source, we set $I_{s}$ equal to 1, and Eq. (1) for the detected image can be rewritten as follows:

2 $$ I\left( {x,y} \right) = I_{0} \left( {x,y} \right)\left( {1 - V\left( {x,y} \right)} \right) + V\left( {x,y} \right). $$

The enhanced image *E* (i.e., the collected image $I,$ once enhanced to restore original intensity $I_{0}$) can be solved as:

3 $$ E\left( {x,y} \right) = \left( {I\left( {x,y} \right) - V\left( {x,y} \right)} \right)/\left( {1 - V\left( {x,y} \right)} \right). $$

The foggy veil *V* can be computed by following the no-black-pixel constraint^13^ and by assuming that *V* has an intensity greater than or equal to zero. A minimal component image $I_{min}$ was calculated from the original image *I* by taking the minimum value of the RGB color channels at each pixel position. The no-black-pixel constraint assumes that the standard deviation of $I_{min}$ within a local window, $std\left( {I_{min} } \right),$ is lower than the average intensity within that window $\overline{{I_{min} }}$, which is described in Eq. 4. To calculate V, a constant factor, *POR* (percentage of restoration), was chosen as a measure of the strength of enhancement to apply to the image $I$, where 99% suggests maximum enhancement.

4 $$ V\left( {x,y} \right) = POR*{\text{max}}\left( {\min \left( {I_{min} \left( {x,y} \right), \overline{{I_{min} }} - std\left( {I_{min} } \right)} \right), 0} \right). $$

Notably, use of too high of a POR for BLC images leads to saturation, unnatural appearance and false-positive fluorescence caused by discoloration. Thus, to identify the optimal POR appropriate for a given BLC image, we added a saturation constraint in the enhancement process, implemented as follows. 

**Figure 5:**
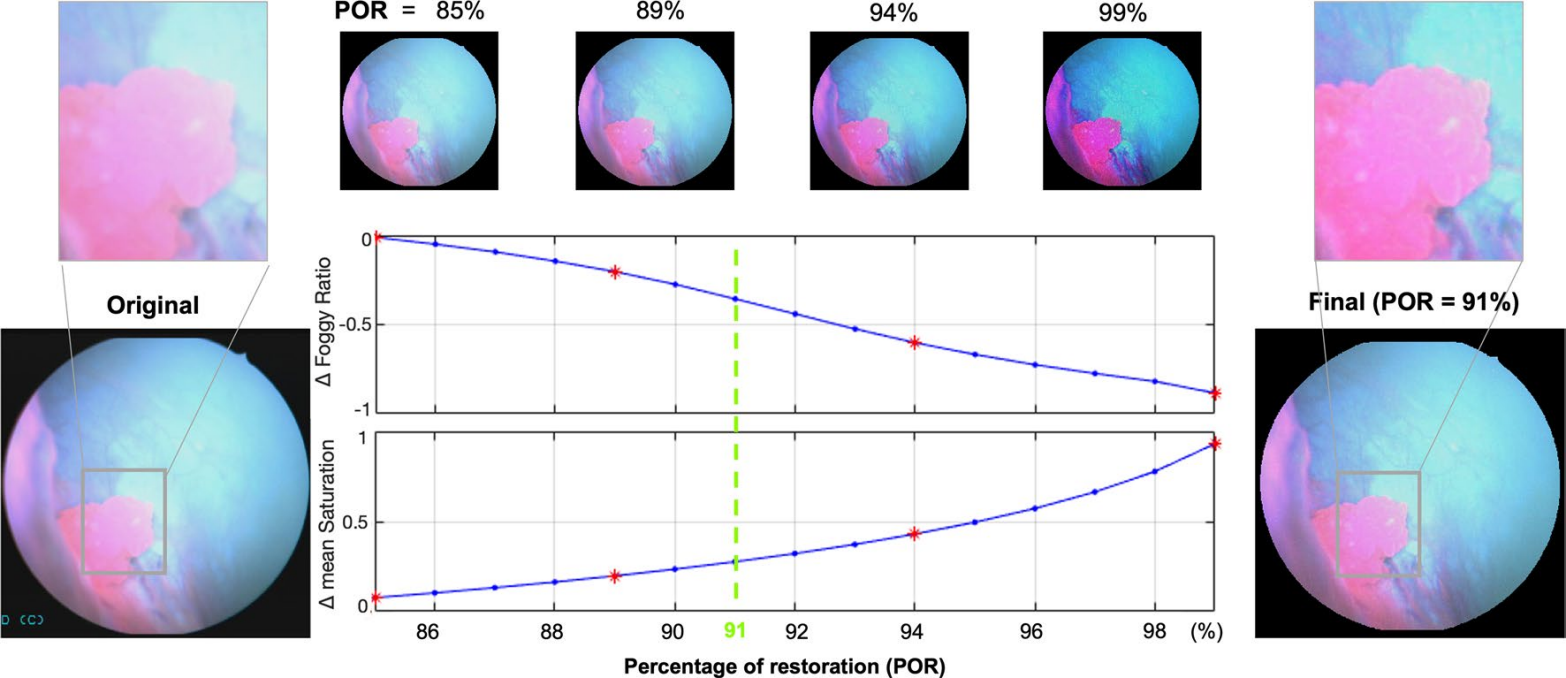
Effect of POR on two key metrics for fog correction: foggy ratio and mean saturation (shown as a difference from the original image value). Note how too high of a correction leads to high saturation. Red asterisks indicate example images (shown above the plots. “Final image” was corrected with a POR of 91%, shown with a green dashed line on the plot.

For each image, we iteratively performed fogginess enhancement by varying the POR from 85 to 99% (Fig. 5). On each iteration, we calculated the percent change between each restored image and the original image for two variables: the average saturation obtained in the HSV color space and the foggy pixel ratio, a new metric we defined that depicts how foggy an image is by calculating the “foggy area” (i.e., number of foggy pixels) versus the total area (i.e., number of total pixels) of an image. To determine the foggy area, we used the “semi-inverse" foggy area detection method^14^, wherein we defined a foggy pixel as one having a small difference (< 0.5, empirically determined) between the hue channels of the image and its semi-inverse. The optimal POR for a given image was determined to be the value at which the change in the foggy pixel ratio was negative (i.e., the restored image becomes less foggy than original) and the change in average saturation was within 0.25 of the original (i.e., minimal increase in image saturation). If no such condition existed, the POR was set to 95%. For videos analyzed in the study, when calculated for all frames, the optimal PORs were found to have a maximum variation of 3% and a standard deviation of 0.65% within a video. Therefore, for real-time implementation of the fogginess correction, only a few PORs may need to be computed for a given video to account for slight changes in fogginess level during imaging.

### Clinical survey

To assess the perceived quality of the enhanced images and the original images, we recruited six urologists from the Vanderbilt University Medical Center to complete a survey answering three questions regarding the clarity, contrast and confidence to make a clinical decision based on information presented in each image. The survey comprised a five-point Likert scale with increasing consent from (1) strongly disagree to (3) neutral to (5) strongly agree. In this study, cystoscopy videos were on average 60 s each, with approximately 30 s being blue light (frame rate = 30 Hz). Of the 31 patients enrolled in the study, we excluded 20 patients whose videos suffered from suboptimal illumination conditions, excessive bleeding, or surgical tools in the FOV. The remaining 11 patients were included in the survey study. The mean age of the 11 patients is 72.5 with a standard deviation of 8.5 years. Among these patients, 8 were male and 3 were female. The survey images comprised one representative image from each patient video (i.e., 11 original images total) and an additional 17 images generated after performing subsequent partial and full enhancements to yield the 28 frames used in the survey: 11 original images, 12 partially enhanced images (green-hue correction only or defogging only), and 5 fully enhanced images (green-hue correction plus defogging). The total number of images was selected to ensure the survey timeline would be reasonable. The tissue types included in the 28 images contain 14 papillary tumors, 6 flat tumors, 4 inflammation sites and 4 normal-appearing bladder walls. These example frames were chosen because they were representative of the types of cystoscopy video samples collected in the study and contained varying levels of imaging artifacts (green hue and fogginess) that were of interest to the study. The other frames not included in the survey were either containing similar information as the chosen frames, or they were out of focus, or suffering from motion blur.

The chosen images are representative cases of green-hue artifact and fogginess seen in the images collected in the study. The original and enhanced images were shown in a randomized order and the reviewers were blinded to the artifact type. One image was shown at a time (on a single page of a multi-page survey) and the reviewers were not given the opportunity to go back to the previous pages and change their responses. Since each image was evaluated with four questions and by six reviewers, a total of 24 count of responses were recorded per image. For statistical evaluation, the mean and standard deviation of the averaged responses was calculated for each image type (original, green corrected only, defogged only and fully enhanced). 

**Figure 6:**
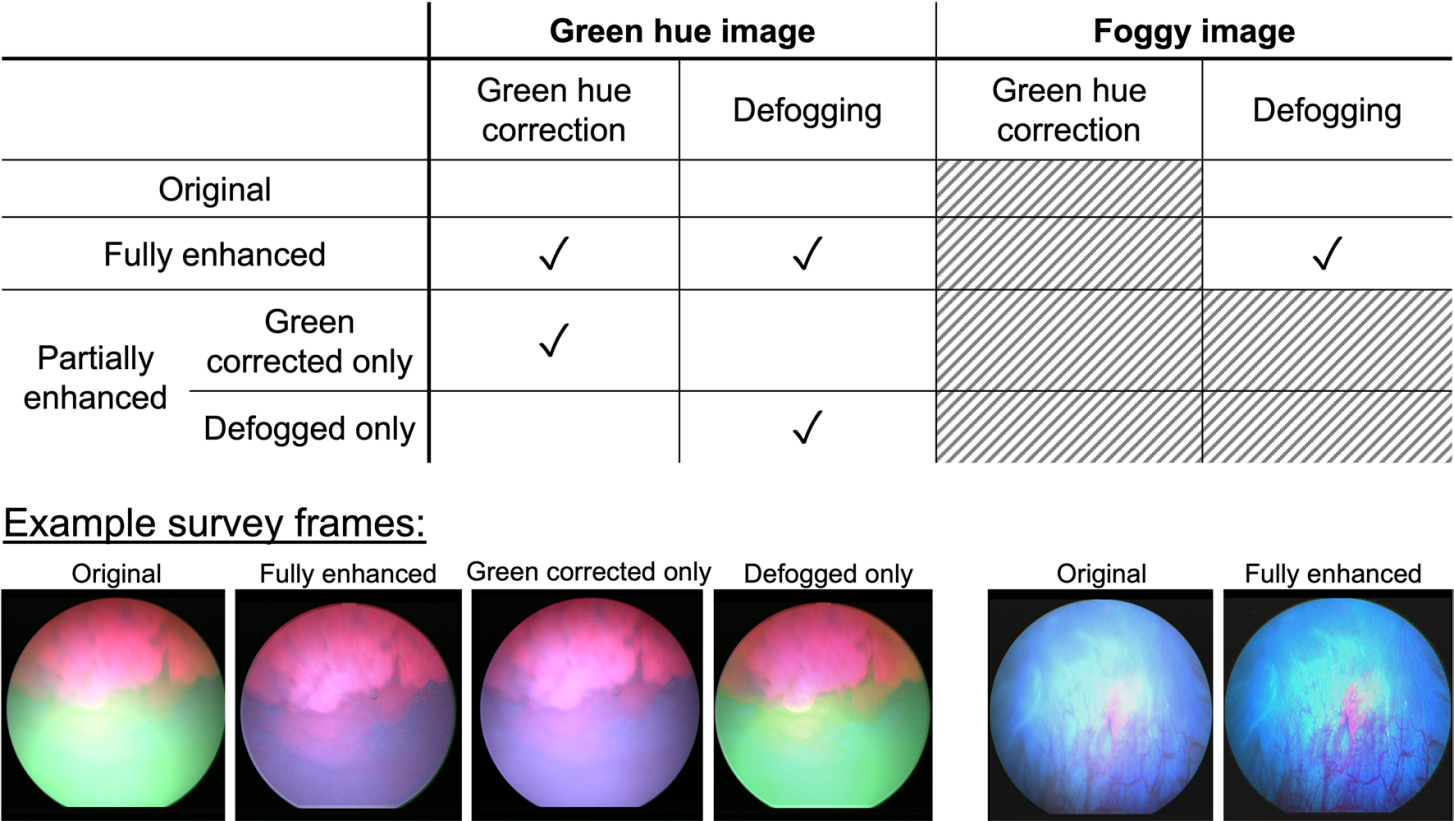
Table of image types and example images included in the survey.

Example images used in the survey are shown in Fig. 6. To evaluate the results, we paired each enhanced image with its original image and used a Wilcoxon matched-pairs signed rank test to compare reviewers’ responses to the original—enhanced image pair and determined the *p* value for each enhancement strategy. From the image pairs, we calculated the difference in the responses for each question and from each reviewer, where a positive value suggests the enhanced image scored favorably over the original image. The differences were averaged across reviewers.

## Results and discussion

**Figure 7:**
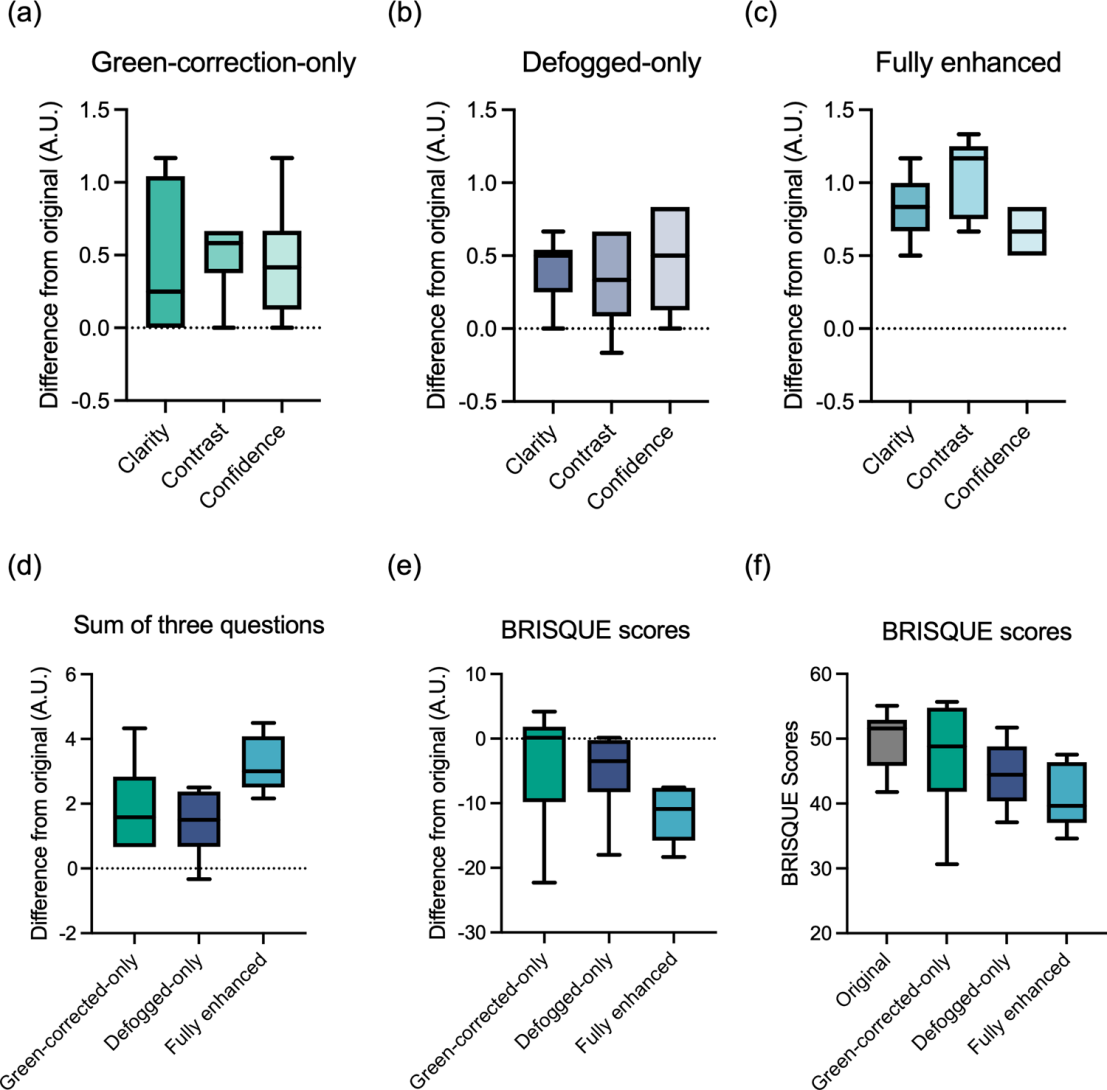
(**a**–**c**) Difference in Likert-scale scores between enhanced and original images (enhanced score minus original score) are shown as box and whisker plots for each question of assessment (clarity, contrast and confidence). (**d**) The summation of Likert scores from the three questions. (**e**) Differences in the BRISQUE scores between enhanced and original images (enhanced score minus original score). (**f**) BRISQUE scores calculated from the original images and each category of enhanced image included in the survey. The whiskers extend to the maximum and minimum values and the line in the box represents the median value.

The qualitative and quantitative results of this study suggest that our enhancements improve the perceptual quality and visualization of images. Survey responses are summarized in Table 1 (mean and standard deviation of reviewer response for each question grouped by image type). To better understand the improvement of scores for each enhanced frame compared to the corresponding original frame, we computed the differences in scores for each enhanced-to-original frame pairs, which are plotted in Fig. 7. Figure 7a–c plots the improvement score for green-corrected, defogged and fully enhanced images for each evaluation category (clarity, contrast and confidence). The sum of the three categories of improvement is also plotted in Fig. 7d. Notably, the green-hue correction step leads to greater contrast improvement (a mean score improvement > 0.5 compared to the original) than the defogging step (shown in Fig. 7a), suggesting that the removal of green-hue artifact allows better visualization of tissue contrast than if only defogging was applied to a green-hued image, and it allows recovery of fluorescence lost due to the presence of urine (i.e., the cause of the green-hue artifact). The defogging step, on the other hand, shows higher scores in the improvement of clarity than the green-hue correction step. However, when a green-hued image undergoes defogging but not green-hue correction, saturation in the green channel is intensified, leading to lower scoring of the enhanced image in the contrast category than the original image. This effect can be seen in the example images in Fig. 6 and the box plots in Fig. 7a**–**d. Both single-step methods result in a similar level of confidence improvement.

**Table 1:** Survey response summary.

Image quality perception	Score (Mean ± SD)			
Score 1–5: “strongly disagree”, “disagree”, “neutral”, “agree”, “strongly agree”	Original	Green corrected only	Defogged only	Fully enhanced
(1) “The image has good clarity”	3.05 ± 0.83	3.19 ± 0.96	3.50 ± 0.71	3.63 ± 0.43
(2) “The image has good contrast”	3.27 ± 0.77	3.39 ± 0.74	3.75 ± 0.71	3.87 ± 0.32
(3) “I am confident to make a clinical assessment using this image”	3.04 ± 0.79	3.33 ± 0.80	3.56 ± 0.80	3.57 ± 0.38
Averaged response	3.11 ± 0.78	3.26 ± 0.77	3.55 ± 0.72	3.63 ± 0.39
*p* value		< 0.0001	< 0.0001	< 0.0001

When both enhancement steps are applied (i.e., images are fully enhanced), the enhanced images score more favorably than images with single-step enhancements and original images. Adding the scores from three evaluation categories, fully enhanced images score more than three points higher than the original images and approximately 1.5 points higher than the images with single step enhancements. The responses from the surveyed urologists suggest the proposed enhancement can provide clearer, better contrasted images that improve diagnostic confidence during cystoscopy imaging. Additionally, although not shown on the graph, the sums of the responses for the three categories of enhanced images are all statistically significantly different from the originals (*p* < 0.0001, with a Wilcoxon matched-pair signed rank test).

To quantitatively evaluate the image enhancement, we computed Blind/Referenceless Image Spatial Quality Evaluator (BRISQUE) scores of the original and enhanced images included in the survey, where better perceptual quality is indicated by a smaller BRISQUE score^15^. Most images achieved lower BRISQUE scores compared to the original, but a few green-hue-corrected-only images had slightly higher BRISQUE scores, as shown in Fig. 7e, f. This is possibly because fogginess remained in those images and BRISQUE scores considered that as a feature of low perceptual image quality. All fully enhanced images had lower BRISQUE scores than their original and reach an average decrease of 21.6%.

**Figure 8:**
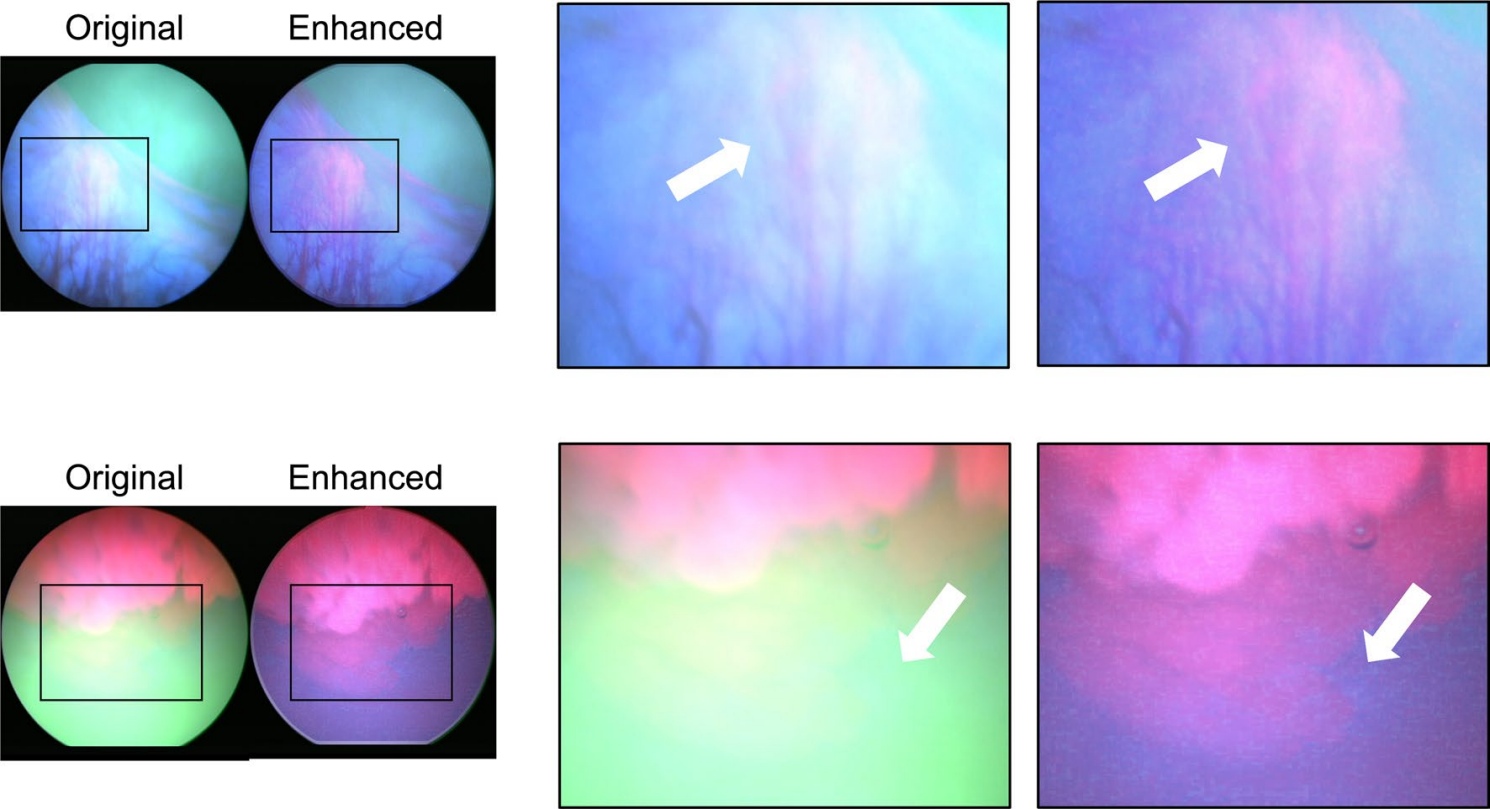
Information recovered through image enhancement reveals important diagnostic features. (**a**) Tumor fluorescence and vascular features are washed out in the original image but recovered in the enhanced image. (**b**) Tumor fluorescence is invisible in the original image and is restored in the enhanced image. White arrows point to the loss of features (in original) and restored features (in enhanced).

It is important to note that the enhancement methods proposed in this study not only result in better clarity and contrast but also recover diagnostic features that are originally lost due to the presence of imaging artifacts. Figure 8 shows two examples of original images that have diminished fluorescence due to green-hue and fogginess. In the corresponding enhanced images, fluorescent features, which are critical in diagnosis, are restored.

**Figure 9:**
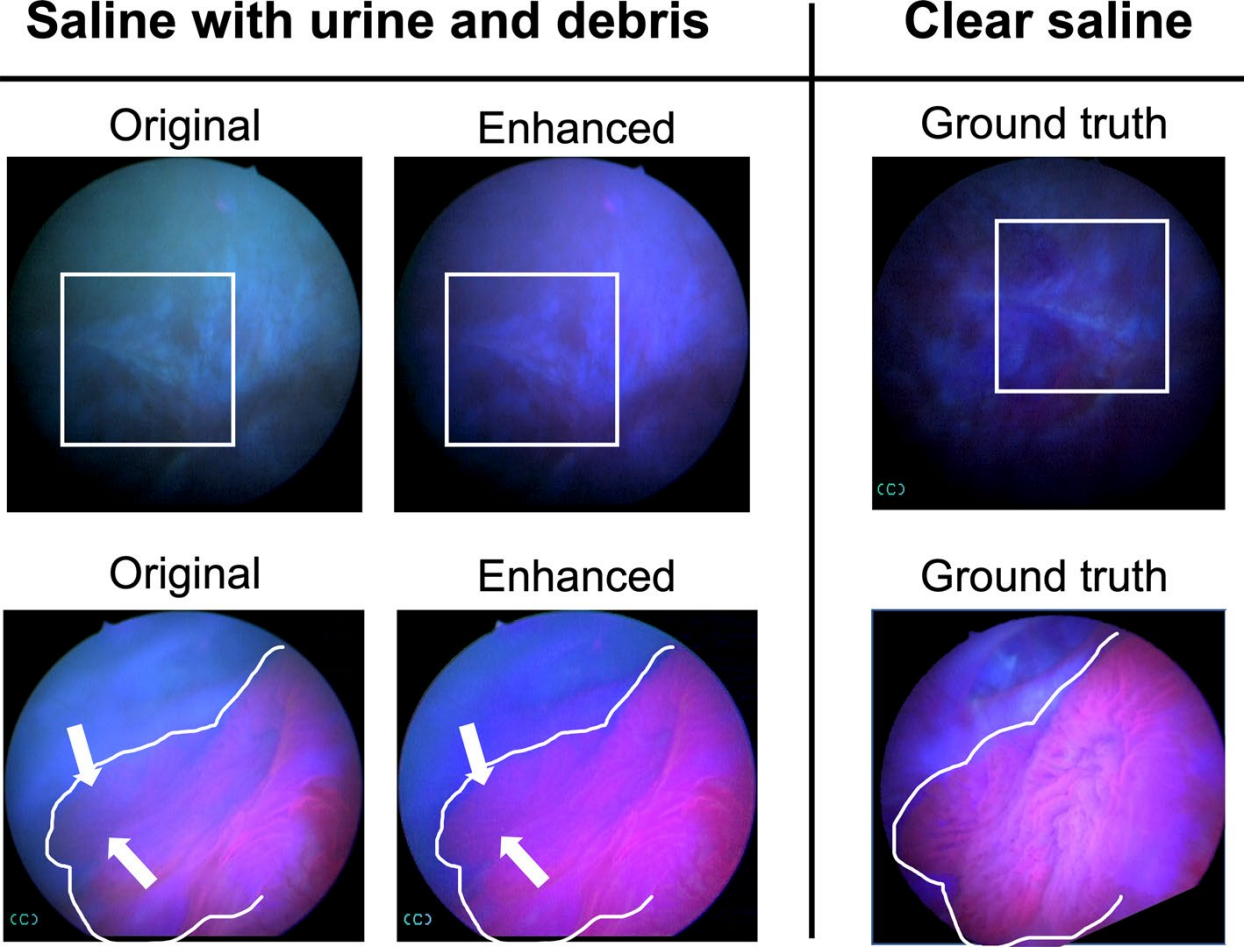
Examples of benign (top row) and cancerous (bottom row) bladder lesions imaged before and after re-distending the bladder with clear saline. When comparing enhanced image to the ground truth image (same tissue region taken at a later time in a clear environment), the enhancement steps preserved fluorescence from cancerous regions (white arrows), without artificially adding false-positive fluorescence to the image. White boxes and outlines show correspondence between the images.

To verify that the fluorescence restored by the enhancement steps is a true representation of tissue fluorescence, we considered videos from example cases where, during the cystoscopy imaging, clinicians emptied the saline in the bladder (with urine or impurities) and re-distended it with clear saline. Videos that contain green-hued and/or foggy portions and re-imaged clear portions of the same bladder region obtained prior and after re-distention, respectively, were analyzed. Images from green-hued and foggy portions were fully enhanced and then compared with images from the clear portion. Figure 9 shows a benign example (top row), where enhancement steps successfully removed the obvious green-hue artifact from the original image and did not introduce false tissue fluorescence, compared with the clear image as the ground truth. Figure 9 also shows a tumor example (bottom row), where diminished fluorescence (see arrows) caused by fogginess in the original image was restored in the enhanced image. The clear, ground truth image shows that the enhancements to the foggy image successfully restored fluorescence, which was previously obscured.

For these two sets of example images, we also computed BRISQUE scores for the original, enhanced and ground truth frames. For the top row (left to right), the BRISQUE scores are 51.56, 49.92, and 50.01, respectively; for the bottom row, the BRISQUE scores are 48.05, 46.01, and 45.84, respectively. Notably, the BRISQUE scores of enhanced frames approach or even outperform the ground truth. In the case of the top row, we suspect the higher BRISQUE scores for the top row may reflect a preference for brighter images, as the ground truth image is clearer but dimmer.

## Conclusion

This study presents the first demonstration of artifact removal for BLC videos. In particular, we focus on removal of green-hue and fogginess artifacts commonly found in BLC video frames. Out of the 45 videos collected in the study, 36% of the videos contain green-hue images and 84% contain foggy images, suggesting the need for a reliable enhancement method to restore the visualization of the bladder wall. The enhancement methods presented in this study allow the effective removal of artifacts in cystoscopy videos while maintaining accurate tissue fluorescence as well as realistic tissue appearance. Survey results and quantitative image quality assessments suggest the enhanced images achieved better perceptual quality compared to the original, which may lead to improved diagnostic confidence of the clinicians during a cystoscopy examination. Importantly, diagnostic features such as tumor fluorescence and vasculature were restored through the enhancement process. We also confirmed that the uncovered tumor fluorescence was not false positive (i.e., algorithm artifacts) by comparing with ground truth images taken in clear environments. 

In the current study, image enhancement methods were performed as post-processing steps in MATLAB (2022a, The MathWorks, Inc.), on a personal computer configured with a 2.6 GHz 6-core Intel Core i7 CPU, 16 GB RAM and a macOS Monterey system. The processing time was 0.274 s per frame during green-hue correction step, 6.322 s per frame for POR computation (15 iterations), and 0.875 s per frame for the defogging step. The most time-consuming step is computation for the optimal POR. To translate the proposed approach clinically, this iterative procedure could be performed once every few minutes, or just a couple of times throughout the procedure, as we observed that the optimal PORs within a single imaging session (if no irrigation was used) had limited variation. Therefore, we also suggest reducing the number of iterations to help speed up the computation. Moving forward to clinical implementation, the computational burden could be further alleviated through algorithm optimization as well as the use of parallel computing with high-performing graphics processing units.

Real-time, automatic corrections to BLC imaging can reduce the operation time and remove the need to remove and refill the bladder with irrigant. As a result, a potential cost savings and reduced risk of morbidity could be achieved through the reduction in the total OR time. Restoration of clear visualization improves confidence in diagnosis and may also lead to more sensitive tumor detection. One limitation of this study is only six urologists were included in the survey. In addition, all enhancements were performed in post-processing and were not implemented in real-time. Nevertheless, the results of this study motivate larger, multi-centered clinical studies to evaluate the clinical implication of the enhancement during real-time imaging or during offline review. 

## Data Availability

Code and data generated in this study can be made available from the corresponding author on reasonable request.
